# Description of *Boleodorus Bushehrensis* n. Sp. (Rhabditida: Tylenchidae) from Southern Iran, and Observations on a Commonly Known Species

**DOI:** 10.2478/jofnem-2022-0004

**Published:** 2022-04-17

**Authors:** Somayeh Monemi, Mohammad Reza Atighi, Joaquín Abolafia, Ebrahim Pourjam, Majid Pedram

**Affiliations:** 1 Department of Plant Pathology, Faculty of Agriculture, Tarbiat Modares University, Tehran, Iran; 2 Departamento de Biología Animal, Biología Vegetal y Ecología, Universidad de Jaén, Campus Las Lagunillas, s/n, 23071, Jaén, Spain

**Keywords:** *Boleodorus thylactus*, LSU rDNA D2-D3, SEM, SSU rDNA, taxonomy

## Abstract

A new species of the genus *Boleodorus*, recovered from southern Iran, is described and illustrated based upon morphological and molecular data. *B. bushehrensis* n. sp. is mainly characterized by having a wide and low cephalic region (which is continuous with the adjacent body), the oral aperture in a depression in side view under a light microscope, four lines in the lateral field, weak metacorpus with a vestigial-to-invisible valve, and short (26–38 mm long) hooked tail with rounded tip. The females are 334–464 mm long and have a spherical spermatheca filled with spheroid sperm; males have 11.5- to 12.0-mm-long spicules and weakly developed bursa. The new species has an annulated low cephalic region, four large cephalic papillae, and small crescent-shaped amphidial openings when observed by scanning electron microscopy (SEM). Its morphological and morphometric differences with seven known species are discussed. The phylogenetic relationships of the new species with other relevant genera and species have been studied using partial sequences of small and large subunit ribosomal DNA (SSU and LSU rDNA). In both the SSU and LSU phylogenies, the sequences of *B*. *bushehrensis* n. sp. and other *Boleodorus* spp. formed a clade. A second species, *B. thylactus*, when studied under SEM, has a raised, smooth cephalic region, four large cephalic papillae, and oblique amphidial slits, with the oral opening in a depression.

The subfamily Boleodorinae Khan, 1964 belongs to the family Tylenchidae Örley, 1880 and includes eight valid genera (Geraert, 2008; Yaghoubi *et al*., 2016; Amiri Bonab *et al*., 2021). Currently, *Boleodorus* Thorne, 1941 represents the largest genus of the subfamily and contains 33 species (Munawar *et al*., 2021), all of which were described using traditional, morphology-based methods. To our knowledge, molecular data and scanning electron microscopic (SEM) images of the type species and type populations of the currently known species are not available. Although Brzeski and Sauer (1982) and Shokoohi (2021) have presented SEM images of two species of the genus. So far, seven species of the genus *Boleodorus* have been reported from Iran (Karegar, 2018).

During a faunistic survey of nematodes associated with palm trees and grasses in southern Iran, a population of *Boleodorus* was recovered. Morphological and morphometric studies showed that it belongs to an unknown species. A population of *Boleodorus thylactus* Thorne, 1941 was also recovered from Golestan Province, northern Iran. Thus, the present study aims to describe the new species using an integrative approach and characterize both species using SEM observations.

## Materials and methods

### Sampling, nematode extraction, and morphological study

In January 2021, 40 soil samples were collected from the east of Bushehr Province, southern Iran. The soil sample containing *B. thylactus* was collected from Golestan Province, northern Iran. Nematodes were extracted from the soil using the tray method (Whitehead and Hemming, 1965) and handpicked under a Nikon SMZ1000 (Nikon, Tokyo, Japan) dissecting microscope. Specimens were heat-killed by adding hot 4% formalin solution, transferred to anhydrous glycerin according to De Grisse (1969), and mounted on permanent slides. Measurements were performed and drawings created using a drawing tube attached to a Nikon E600 (Nikon, Tokyo, Japan) light microscope and were redrawn using CorelDraw software version 2020. Digital images of the fresh individuals and mounted specimens were taken with Olympus DP72 digital camera (Olympus, Tokyo, Japan) attached to an Olympus BX51 microscope (Olympus, Tokyo, Japan) powered with differential interference contrast optics. The measured indexes and calculated ratios were according to Siddiqi (2000).

### SEM analysis

Three females of each species mounted in glycerin were selected for SEM observations. The nematodes were hydrated in distilled water, dehydrated in a graded ethanol–acetone series, critical point-dried, coated with gold, and observed with a Zeiss Merlin scanning electron microscope (5 kV) (Zeiss, Oberkochen, Germany) (Abolafia, 2015).

### DNA extraction, PCR, and sequencing

For molecular phylogenetic studies of the new species, eight live females were selected, observed in a drop of clean water, washed, and photographed. Each specimen was transferred to a small drop of Tris–EDTA (TE) buffer (10 mM Tris-Cl, 0.5 mM EDTA; pH 9.0, Qiagen, Hilden, Germany) on clean slides and squashed using a clean cover glass. Each suspension (DNA sample) was retrieved by adding 15 μl TE buffer and stored at −20 °C. Primers for small subunit (SSU) ribosomal DNA (rDNA) amplification included the forward primer 22F (5′-TCCAAGGAAGGCAGCAGGC-3′) and the reverse primer 13R (5′-GGGCATCACAGACCTGTTA-3′) (Dorris *et al*., 2002), the forward primer 1813F (5′-CTGCGTGAGAG GTGAAAT-3′), and the reverse primer 2646R (5′-GCTACCTTGTTACG ACTTTT-3′) (Holterman *et al*., 2006). Primers for the large subunit (LSU) rDNA D2-D3 amplification included the forward primer D2Tyl (5′-GAGAGAGTTAAANAGBACGTG-3′) (Oliveira *et al*. 2013) and the reverse primer 1006R (5′-GTTCGATTAGTCTTTCGCCCCT-3′) (Holterman *et al*., 2008). The PCR mixture (30 μl) contained the following: 15 μl *Taq* DNA Polymerase 2× Master Mix RED, 2-mM MgCl_2_ (Ampliqon, Odenese, Denmark), 8 μl distilled water, 1 μl of each primer, and 5 μl of DNA template. The thermocycling program for amplification of both loci was as follows: denaturation at 95°C for 4 min, followed by 32 cycles of denaturation at 94°C for 30 sec, annealing at 52°C for 40 sec, and extension at 72°C for 80 sec. A final extension was performed at 72°C for 10 min. The PCR products were sequenced in both directions using the same primers with an ABI 3730XL sequencer (Bioneer Corporation, Daejeon, South Korea). The newly generated sequences were deposited into the GenBank database under the accession numbers OK018183 for SSU, and OK018176 and OK018177 for LSU rDNA D2-D3.

### Phylogenetic analyses

The chromatograms of DNA sequences were checked using Chromas Lite 2.1.1 (http://technelysium.com.au/), edited/trimmed, and assembled manually. Both SSU and LSU sequences were then compared with other available sequences in the GenBank database using the basic local alignment search tool (BLAST). Several sequences of representatives of the family Tylenchidae were selected for both SSU and LSU phylogenies. Representatives of Aphelenchoidea Fuchs, 1937 were used as outgroups in both phylogenies. In total, 84 SSU and 116 LSU sequences (including newly generated sequences of the new species and aphelenchoidid sequences as outgroups) were included in the SSU and LSU phylogenies (for accession numbers, see the trees). The Q-INS-i algorithm of the online version of MAFFT (version 0.91b) (https://mafft.cbrc.jp/alignment/server/) (Katoh and Standley, 2013) was used to align the SSU data set, and the resultant alignment was manually edited using MEGA6 (Tamura *et al*., 2013). The LSU data set was aligned using ClustalX2 (http://www.clustal.org/), and the resultant alignment was manually edited using MEGA6 (Tamura *et al*., 2013). The best-fitting substitution model for both data sets was selected using PAUP*/MrMod-eltest.2 (Nylander, 2004). The Akaike-supported model, a general time-reversible (GTR) model, including among-site rate heterogeneity and estimates of invariant sites (GTR + gamma [G] + invariant [I]), was selected and used in both phylogenies. Bayesian analysis was performed using MrBayes 3.1.2 (Ronquist and Huelsenbeck, 2003), by running the chains (two chains, as default) for 5 × 10^6^ generations for both data sets. After discarding burn-in samples, the remaining samples were retained for further analyses. The Markov chain Monte Carlo (MCMC) method within a Bayesian framework was used to estimate the posterior probabilities of the phylogenetic trees (Larget and Simon, 1999) using the 50% majority rule. Convergence of model parameters and topology were assessed based on average standard deviation of split frequencies and potential scale reduction factor values. Adequacy of the posterior sample size was evaluated using autocorrelation statistics as implemented in Tracer v.1.6 (Rambaut and Drummond, 2009). The output files of the trees were visualized using Dendroscope v3.2.8 (Huson and Scornavacca, 2012) and drawn in CorelDRAW software version 2020.

## Results

### *Boleodorus bushehrensis* n. sp.

**Figure 1:**
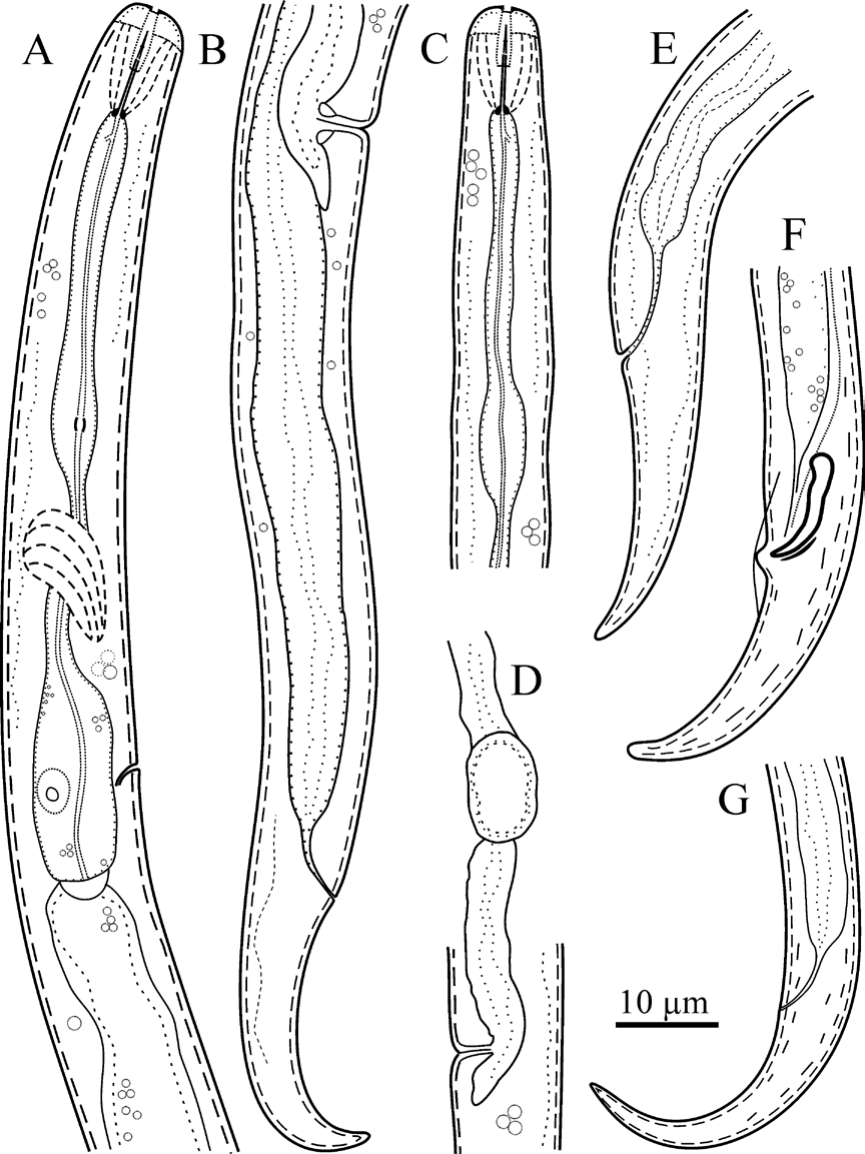
Line drawings of *Boleodorus bushehresnsis* n. sp. (A, B, D, E, G: female; C, F: male). (A) Pharynx. (B) Posterior body region. (C) Anterior body region. (D) Vulval region, showing offset spermatheca. (E–G) Tail.

**Figure 2:**
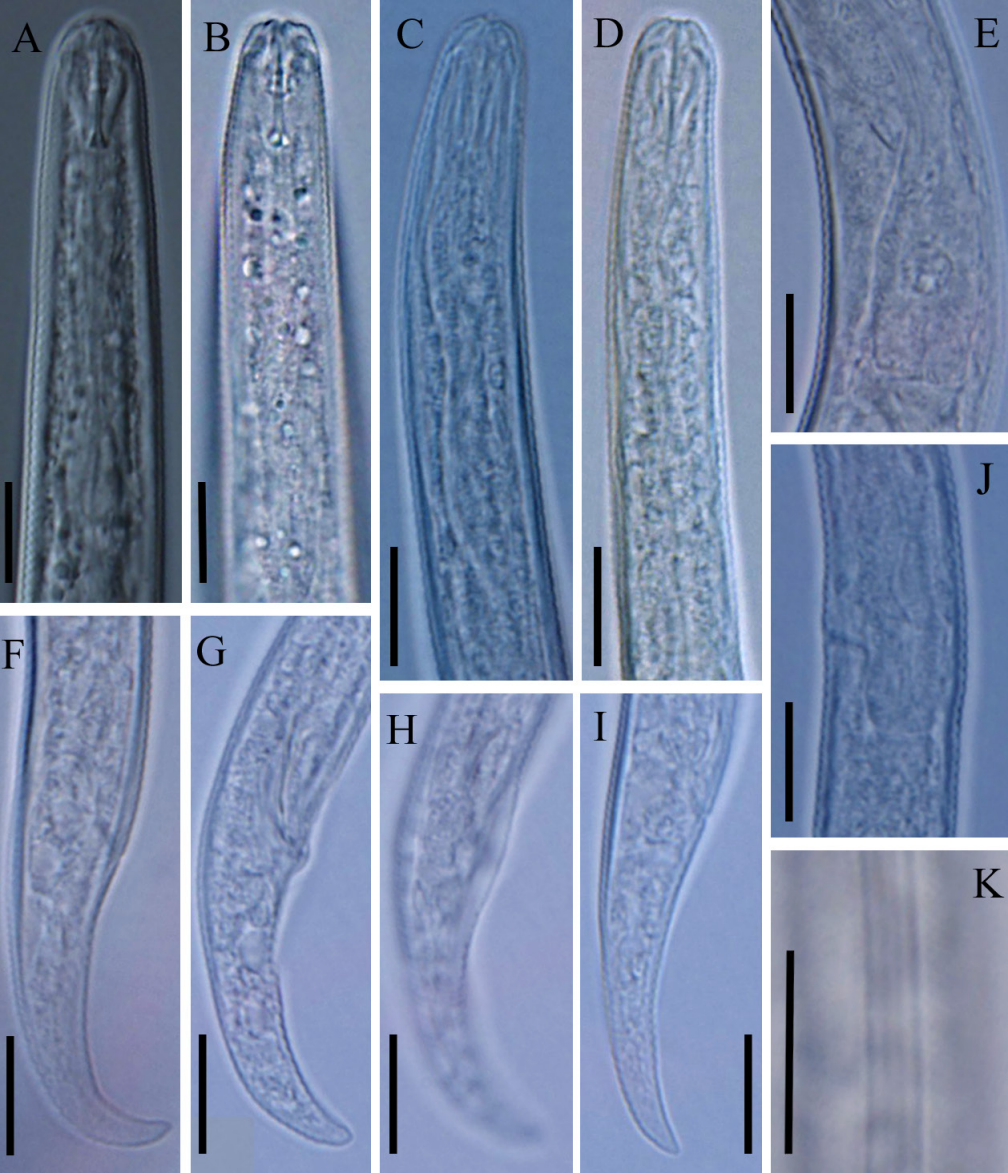
Light microphotographs of *Boleodorus bushehrensis* n. sp. (A, B, C, E, F, I, J, K: female; D, G, H: male). (A, B) Anterior region showing cephalic region and stylet, respectively. (C) Pharyngeal metacorpus. (D) Anterior body region showing the oral aperture in a depression. (E, J) Pharyngeal bulb region showing excretory pore. (F, G, I) Tail tip. (H) Bursa. (K) Lateral field at midbody. (All scale bars = 10 μm).

**Figure 3:**
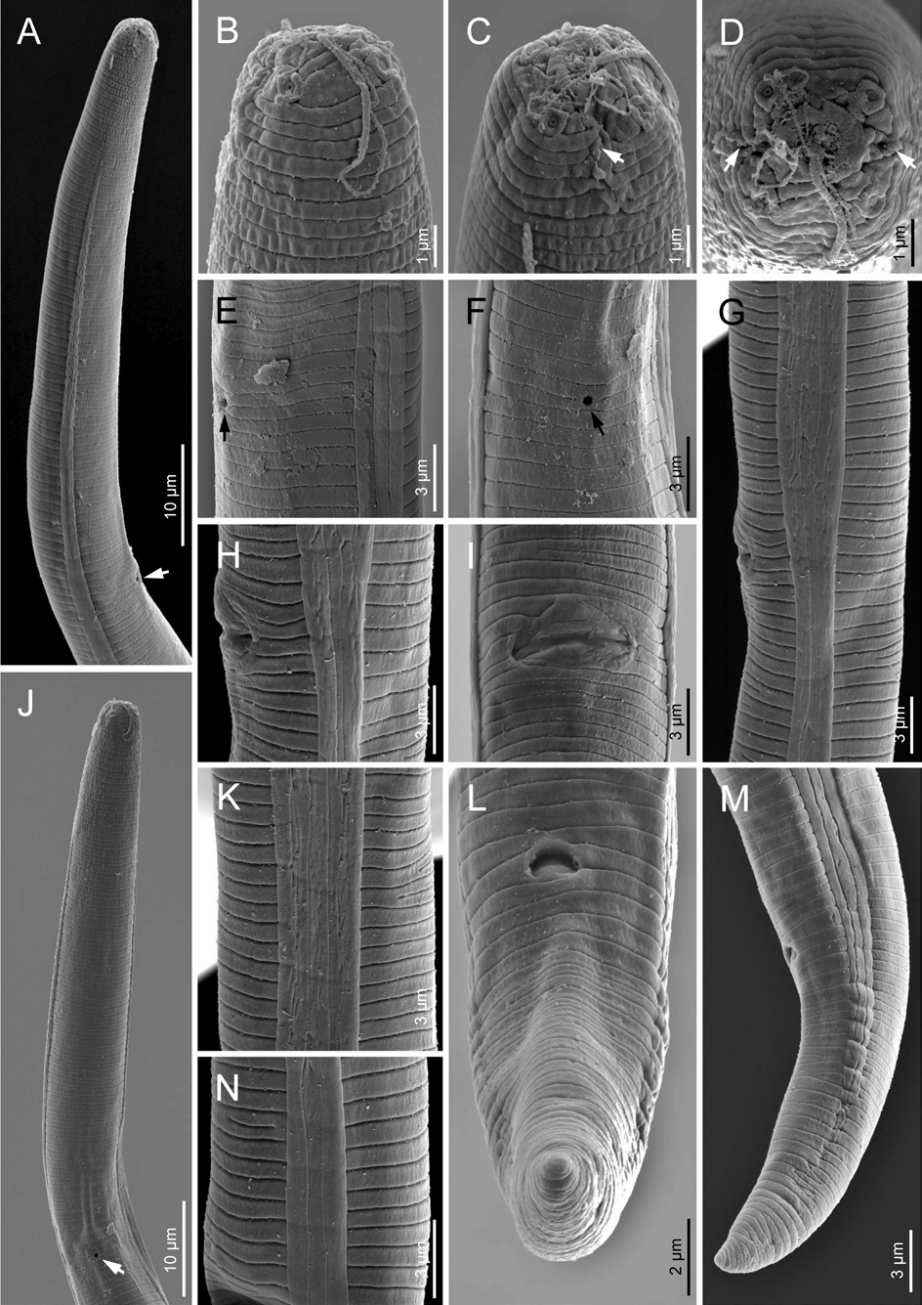
Scanning electron microphotographs of *Boleodorus bushehrensis* n. sp. (female). (A) Anterior body region showing beginning of lateral field (arrow showing the excretory pore). (B–D) Anterior end in ventrolateral, ventral, and *en face* views, respectively (arrows pointing to the amphidial openings). (E, F) Excretory pore in lateral and ventral views, respectively (arrow). (G) Lateral field at vulva. (H, I) Vulva in lateral and ventral views, respectively. (J) Anterior body region in ventral view showing excretory pore. (K,N) Lateral field at midbody showing unusual division and four incisures, respectively. (L,M) Anus in ventral and lateral views, respectively.

The morphological characters of *B. bushehrensis* n. sp. are represented in Figures 1–3.

For the measurements of *B. bushehrensis* n. sp., see Table 1.

**Table 1:** Morphometrics of *Boleodorus bushehrensis* n. sp.

**Characteristics**	**Holotype**	**Paratypes**	
		**Females**	**Males**
n		13	3
L	449	412 ± 35.5 (334–464)	432 ± 17 (417–450)
L’	413	379 ± 34 (298–432)	399 ± 16 (385–416)
a	34	32.5 ± 3.0 (27.8–37.4)	39.8 ± 2.5 (37.2–42.0)
b	4.8	4.8 ± 0.5 (4–6)	5.0 ± 0.5 (4.3–5.4)
c	12	12.6 ± 1.5 (9.3–15.8)	13.1 ± 0.1 (12.9–13.0)
c’	4.9	3.9 ± 0.5 (2.9–4.9)	4.1 ± 0.1 (4.0–4.3)
V or T	79	74.8 ± 1.9 (70.8–79.5)	–
V’	80.7	80.9 ± 1.2 (77.5–82.0)	–
Cephalic region height	2.3	2.1 ± 0.3 (1.7–2.6)	2.1 ± 0.4 (1.8–2.7)
Cephalic region width	6.5	5.9 ± 0.6 (5.0–6.7)	6.1 ± 0.4 (5.6–6.5)
Stylet length	8	8.7 ± 0.4 (8.0–9.3)	9.7 ± 0.3 (9.5–10.0)
Conus length	3	3.2 ± 0.2 (3.0–3.5)	3.3 ± 0.3 (3.0–3.5)
m	37.5	36.8 ± 3.4 (32.3–42.7)	33.6 ± 2.3 (31.5–36.0)
DGO	3	3.1 ± 0.2 (2.8–3.5)	3.0 ± 0.07 (2.9–3.0)
Distance from excretory pore to anterior end	78.5	69.5 ± 5.2 (64–79)	63.9 ± 1.6 (62–65)
MB	48.2	55.8 ± 12.1 (43.0–82.3)	57.8 (n=1)
Pharynx length	91	87 ± 7 (73–99)	87.7 ± 7.5 (80–95)
Distance from anterior end to vulva	333	307 ± 28 (245–347)	–
BW	12	12.7 ± 1.3 (12–15)	10.8 ± 0.3 (10.6–11.0)
Anal BW	9	8.6 ± 0.6 (7–9)	8.7 ± 0.9 (8–9)
Vulva–anus (V–A) distance	80	72.2 ± 8.5 (54–84)	–
PUS length	11.5	10.6 ± 1.3 (8.2–12.0)	–
Tail/V–A	0.5	0.5 ± 0.07 (0.4–0.7)	–
PUS/BW	1	0.8 ± 0.07 (0.7–1.0)	–
Tail length	36	33 ± 3 (26–38)	32.5 ± 0.7 (32–33)
Spicules length	–	–	11.5 ± 0.4 (11.5–12.0)
Gubernaculum length	–	–	4.5 ± 0.2 (4–5)
Bursa length	–	–	6.3 ± 0.5 (6–7)

All measurements are in micrometers and in the form: mean ± SD (range).

BW, body width; DGO, dorsal gland opening; PUS, postvulval uterine sac; SD, standard deviation.

## Description

### Female

Body is short, open C-shaped after fixation, gradually narrowing toward both extremities. Cuticle with fine, transverse annules, being stronger at the distal region of the tail. Lateral fields show four lines, sometimes with irregular additional lines under SEM; the outer lines are plain. Cephalic region low and continuous with the adjacent body. The SEM and light microscopic observations show that the oral aperture is placed in a depression. SEM images further revealed that the cephalic region is annulated; the amphidial openings are short crescent-shaped slits, and four large cephalic papillae are present. Stylet is fine, the conus is shorter than the shaft, and the knobs are small and posteriorly directed. The pharyngeal dorsal gland orifice (DGO) is positioned posterior to the knobs at less-than-half-stylet length. Pharyngeal procorpus is slender; metacorpus is slightly swollen, with vestigial-to-invisible valve plates in the shape of two small roads; isthmus is narrow and slender; and the pharyngeal bulb is pyriform, with usually one visible nucleus. The excretory pore is located at the middle of the pharyngeal bulb or in a position slightly anterior to it. The hemizonid is indistinct. The nerve ring surrounds the anterior part of the isthmus; the cardia is small and hemispherical. Intestine is simple; rectum and anus are functional. The reproductive system is monodelphic–prodelphic, composed of an outstretched ovary, oocytes arranged in a single row, a poorly discernible apparently tubular oviduct, and rounded, offset, and functional spermatheca filled with spheroid sperm cells. Crustaformeria is with unclear cell arrangement; the uterus is simple, and the vagina is perpendicular to the body axis, straight or slightly anteriorly sloping. The postvulval uterine sac (PUS) is about as long as the vulval body diameter. Tail is conical, and usually, its distal region is ventrally curved, forming a hook; in some specimens, it is concave on the ventral side, and its tip is finely or widely rounded.

### Male

Males are rare and functional (sperm observed inside females’ spermatheca). They are similar to females in general morphology, except in sexual characters. Spicules are tylenchoid, slender, and arcuate. Gubernaculum is simple and small. Tail is similar to that of females. Bursa is adcloacal and small, with smooth margin.

### Diagnosis and relationships

The new species is mainly characterized by having a wide and low cephalic region continuous with the adjacent body, annulated in SEM observations; the oral aperture is in a depression in both light and SEM microscopy studies. Short crescent-like amphidial slits are found on SEM analysis; four lines are present in the lateral field, and tail is hooked, short, 26- to 38-µm long with a rounded tip. The new species is further characterized by having 334- to 464-μm-long females, a weakly developed metacorpus with vestigial-to-invisible valve, and offset spherical spermatheca filled with spheroid sperm; males are present, having 11.5- to 12.0-μm-long spicules and weakly developed bursa. By having a wide, continuous, and annulated cephalic region and short tail, the new species is unique in the genus. It is compared with seven known species of the genus having a conical tail and four lines in the lateral field, namely, *B. acutus* Thome and Malek, 1968, *B. azadkashmirensis* Maqbool, Shahina, and Firoza, 1990, *B. citri* Edward and Rai, 1970, *B. cynodoni* Fotedar and Mahajan, 1974, *B. modicus* Lal and Khan, 1988, *B. neosimilis* Geraert, 1971, and *B. volutus* Lima and Siddiqi, 1963. The comparison of the new species with the aforementioned species is as follows.

Compared to *B. acutus,* it has a shorter body length (412 [334–464] μm vs 500 μm), wide and low cephalic region (vs narrower and higher, according to the original drawings), shorter stylet (8.7 [8.0–9.3] μm vs 13 μm), shorter pharynx (87 [73–99] μm vs 114 μm), greater c value (12.6 [9.3–15.8] vs 8), greater V value (74.8 [70.8–79.5] vs 67), and shorter tail (33 [26–38] μm vs 63 μm).

It differs from *B. azadkashmirensis* by the presence of a continuous, wide, and low cephalic region (vs narrower and high), shorter stylet (8.7 [8.0–9.3] μm vs 10.5–12.0 μm), presence vs absence of a vestigial valve in the median bulb, a shorter pharynx (87 [73–99] μm vs 96 μm), greater V value (74.8 [70.8–79.5] vs 63.5–67.5), greater c value (12.6 [9.3–15.8] vs 7–8), and shorter tail (33 [26–38] μm vs 60 μm) not ending in a ventrally curved tip (vs ventrally curved).

It can be distinguished from *B. citri* by having a C-shaped (vs spiral) longer body (412 [334–464] μm vs 280–310 μm), wide and lower cephalic region (vs narrow and higher, according to the original drawings), shorter stylet (8.7 [8.0–9.3] μm vs 9.0–10.5 μm), posteriorly located excretory pore (69.5 [64–79] μm vs 55–61 μm from anterior body end), greater a value (32.5 [27.8–37.4] vs 21–23), greater MB (55.8 [43.0–82.3] vs 35), greater V value (74.8 [70.8–79.5] vs 64–68), and shorter tail (33 [26–38] μm vs 51–82 μm).

It differs from *B. cynodoni* by the shorter body length (412 [334–464] μm vs 420–490 μm), greater V value (74.8 [70.8–79.5] vs 62–65), and shorter tail (33 [26–38] μm vs 54 μm).

It differs from *B. modicus* by having a C-shaped (vs spiral) body, wide cephalic region at the apex (vs narrower), greater c value (12.6 [9.3–15.8] vs 7.7–9.8), greater V value (74.8 [70.8–79.5] vs 66–71), and shorter tail (33 [26–38] μm vs 51 μm).

It is different from *B. neosimilis* by having a wider cephalic region at the apex (vs narrower), a greater V value (74.8 [70.8–79.5] vs 68), a shorter tail (33 [26–38] μm vs 51 μm), and shorter spicules (11.5 [11.5–12.0] μm vs 14 μm).

The new species differs from *B. volutus* by having a C-shaped body (vs spiral), a slightly shorter body length (412 [334–464] μm vs 390–510 μm), a wider cephalic region at the apex (vs narrower), presence of a vestigial valve in the metacorpus (vs absence), shorter pharynx (87 [73–99] μm vs 92–104 μm), and shorter tail (33 [26–38] μm vs 35–60 μm).

### Type habitat and locality

The new species was recovered from a soil sample collected from the rhizosphere of wheat in Shah Firouz village (south of Dashtestan), Bushehr Province, southern Iran, on 30 January 2021. The global positioning system (GPS) coordinates are 29°32.316′N and 50°54.303′E.

### Type material

Holotype female, 10 to 13 paratype females, and three paratype males were deposited at the Nematology Collection of the Faculty of Agriculture, Tarbiat Modares University, Tehran, Iran.

### Etymology

The specific epithet refers to the Bushehr Province, where the new species was found.

### Molecular phylogenetic analyses

**Figure 4:**
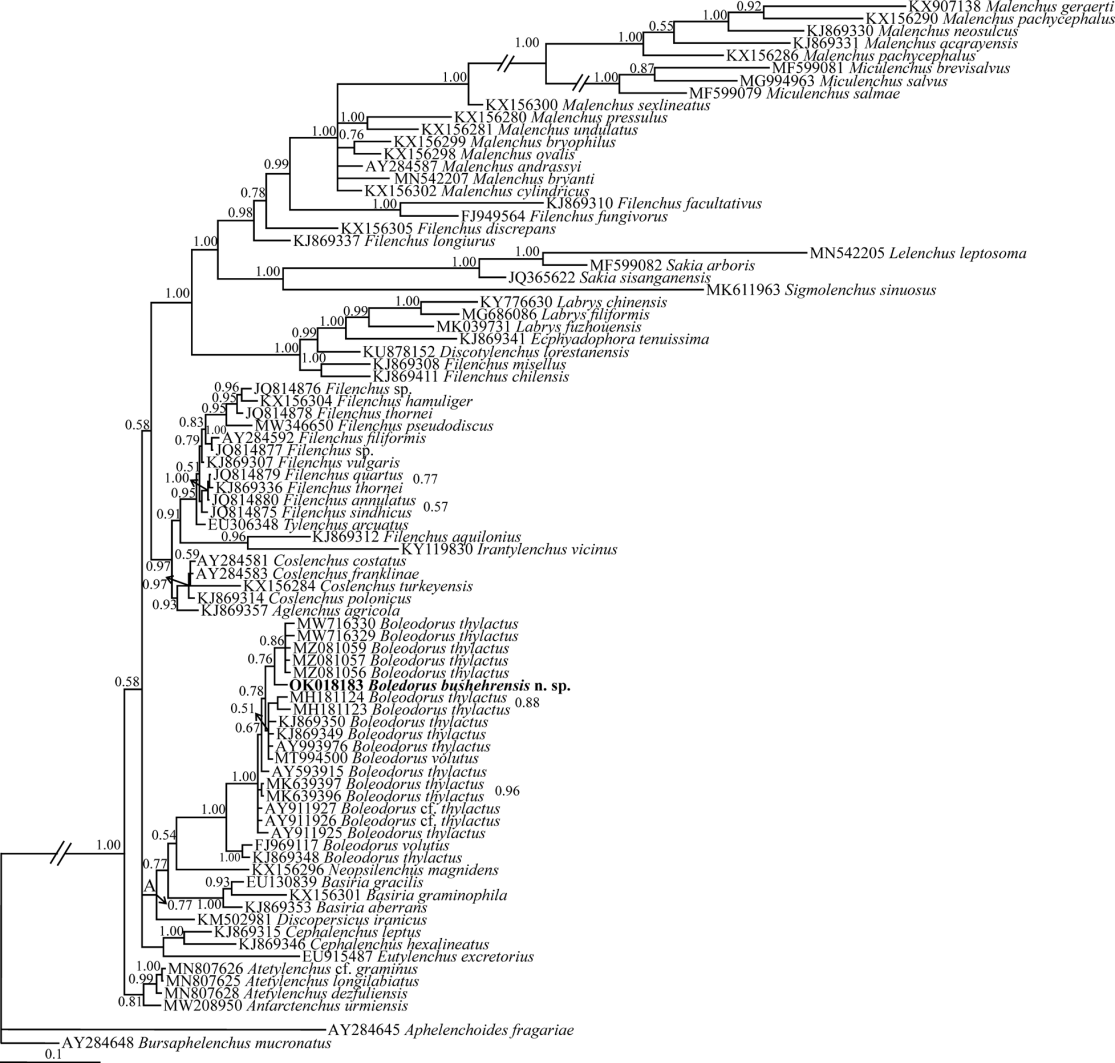
Bayesian 50% majority rule consensus tree inferred from the SSU rDNA of *Boleodorus bushehrensis* n. sp. under the GTR + G + I model. Bayesian posterior probability values are given for corresponding clades. The new species is in bold font. GTR, general time-reversible; G, gamma; I, invariant; rDNA, ribosomal DNA; SSU, small subunit.

Sequencing of the SSU and LSU rDNA D2–D3 fragments of the new species yielded a single 1,242-nt-long SSU (accession number OK018183); and two 517- and 584-nt-long LSU sequences (accession numbers OK018176 and OK018177). The BLAST search using the newly generated SSU sequence revealed a 98.00%–98.79% identity with nine sequences assigned to *B. thylactus* (KJ869348, KJ869350, AY993976, AY593915, KJ869349, MW716329, MK639397, MK639396, and MW716330). Its identity with the sequence assigned to *B. volutus* (FJ969117) was 98%. In the SSU phylogenetic tree (Fig. 4), the DNA sequences of Boleodorinae formed a poorly supported clade (Clade A, 0.77 Bayesian posterior probability [BPP]). The DNA sequences representing *Boleodorus* formed a major clade, and the sequences assigned to *B. thylactus* occupied different placements in this tree. The new species formed a poorly supported clade (0.76 BPP) with five sequences assigned to *B. thylactus* (MW716330, MW716329, MZ081056, MZ081059, and MZ081057).

**Figure 5:**
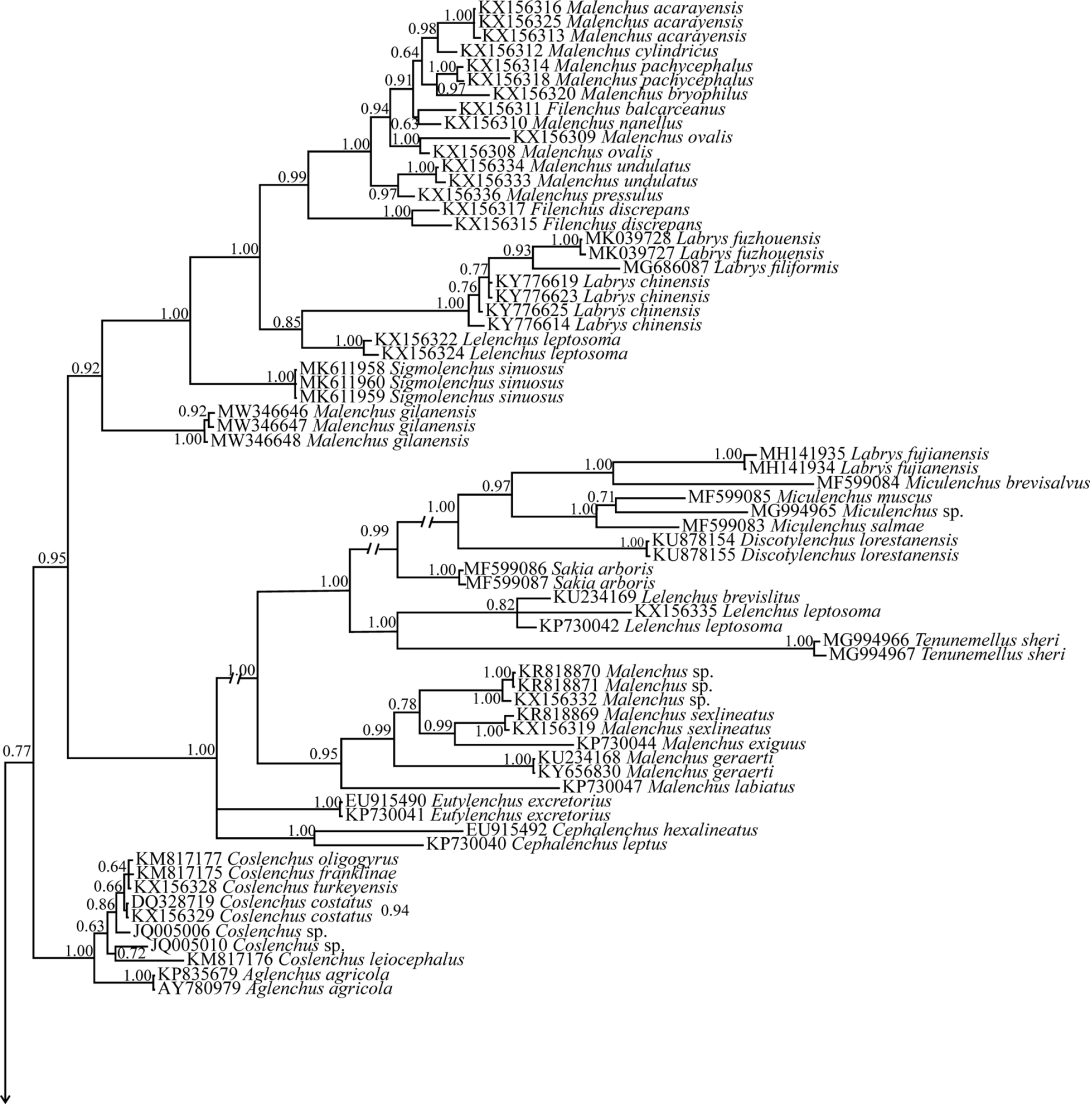

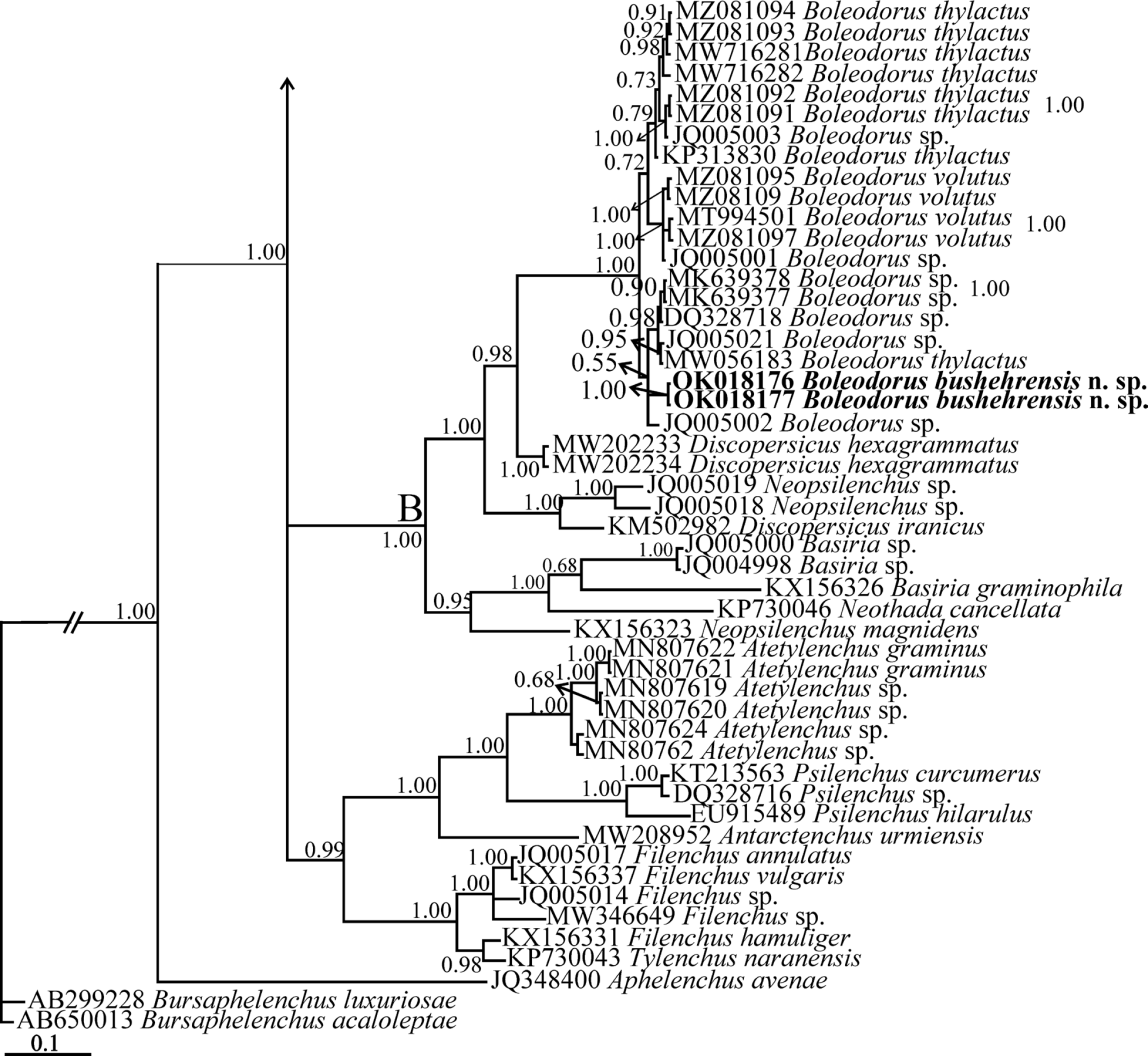
Bayesian 50% majority rule consensus tree inferred from the LSU rDNA D2–D3 sequences of *Boleodorus bushehrensis* n. sp. under the GTR + G + I model. Bayesian posterior probability values are given for the corresponding clades. The new species is in bold font. GTR, general time-reversible; G, gamma; I, invariant; LSU, large subunit; rDNA, ribosomal DNA.

The BLAST search using the LSU sequence of the new species (OK018177) revealed that its identity with all currently available LSU sequences of Tylenchidae is <96% (the highest identity was 95.02%, belonging to *Boleodorus* sp. [JQ005002]). The DNA sequences of Boleodorinae (for phylogenetic status of *Atetylenchus* Khan, 1973, see the Discussion section) formed a maximally supported clade in the LSU tree (Fig. 5, clade B). Sequences of *Boleodorus* formed a clade; however, several sequences assigned to *B. thylactus* occupied different placements. The relationships of the new species with six sequences (JQ005002, MW056183, JQ005021, DQ328718, MK639377, and MK639378) have not been resolved due to polytomy.

### Iranian population of *B. thylactus*

**Figure 6:**
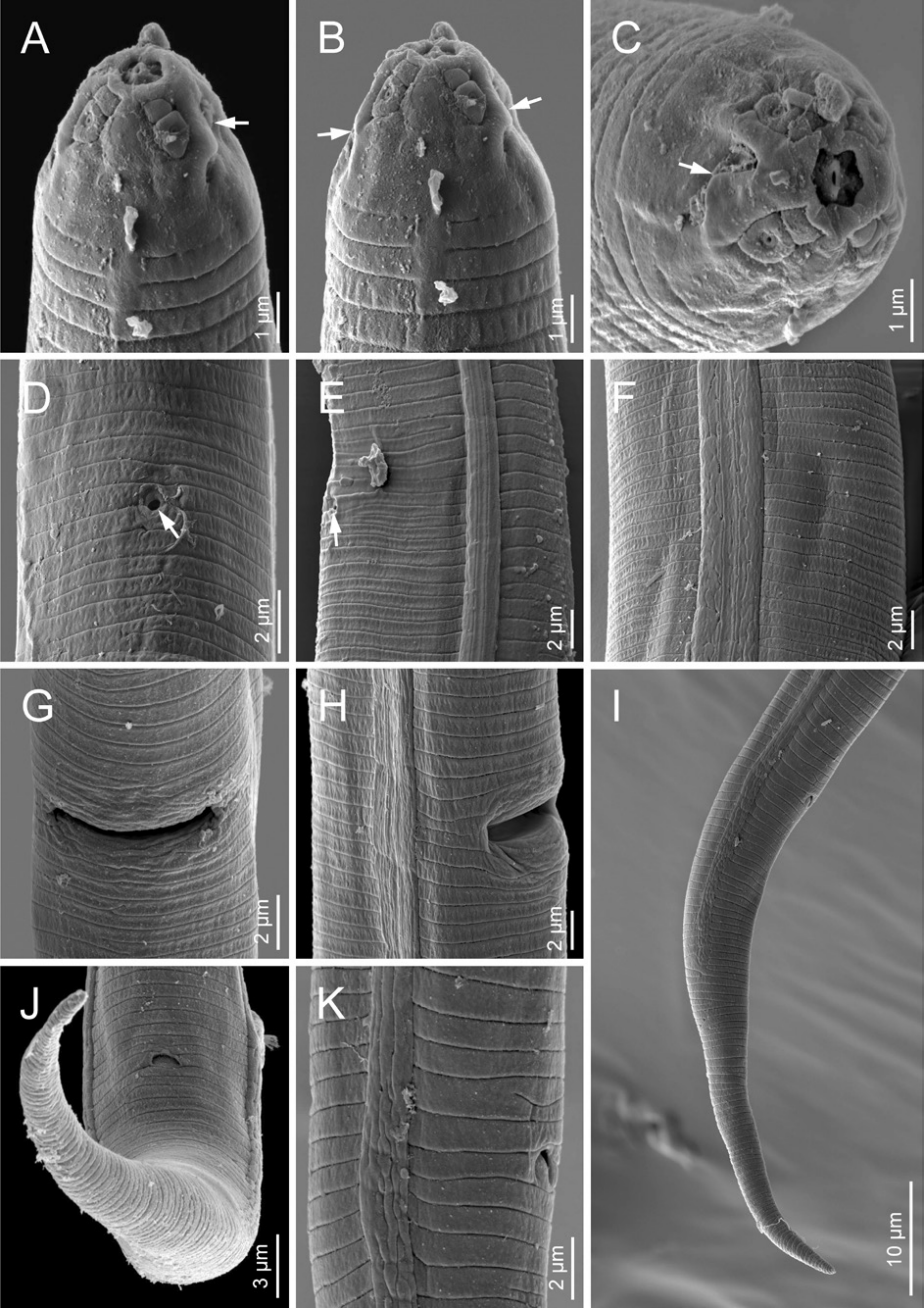
Scanning electron microphotographs of *Boleodorus thylactus* Thorne, 1941 (female). (A–C) Anterior end in ventral and frontal views, respectively (arrows pointing to the amphidial openings). (D,E) Excretory pore in ventral and lateral views (arrow). (F, K) Lateral field at midbody showing four incisures. (G,H) Vulva in lateral and ventral views, respectively. (I) Posterior end in lateral view. (J) Anus in ventral view.

The Iranian population of *B. thylactus* is shown in Figure 6.

The presently studied population of *B. thylactus* was recovered from Golestan Province, northern Iran (GPS data: 37°8.476′N, 55°21.394′E), in association with unidentified forest trees, and its morphological and morphometric data were in full agreement with the data given by Geraert (2008).

## Discussion

SEM has a pivotal role in taxonomic studies of Tylenchidae. In recent studies, the identities of several genera and species have been clarified using this information (e.g., Panahandeh *et al*., 2018; Qing and Bert, 2018; Panahandeh *et al*., 2019a, 2019b, 2019c; Gharahkhani *et al*., 2020; Mortazavi *et al*., 2021). The present SEM observations revealed that the cephalic region pattern of the two studied species is in agreement with the data presented by Brzeski and Sauer (1982) and the type VI of cephalic region pattern of Tylenchidae depicted by Geraert and Raski (1987) and Qing and Bert (2018), i.e., the cephalic papillae are large and the amphidial openings are oblique slits. According to Geraert (1971), the cephalic region of *Boleodorus* spp. could be low and flat, to conical and high. The SEM observations of the Iranian population of *B. thylactus* corroborated that the cephalic region is high, conical, and smooth (in accordance with Brzeski and Sauer, 1982), and that the other features are in accordance with the description of the cephalic region for the genus provided by Siddiqi (2000). The SEM observations of the new species provided new characteristics for the cephalic region for *Boleodorus*: the cephalic region is annulated, and the amphidial openings are short and appear as crescent-like slits.

In the SSU- and LSU-based phylogenetic trees, representatives of Boleodorinae formed a clade (although with poor support in the SSU phylogeny) except for the genera *Psilenchus* De Man, 1921 and *Atetylenchus* (Boleodorinae *sensu* Geraert, 2008, well suitable to be placed under Psilenchidae Paramonov, 1967, see Amiri Bonab *et al*., 2021). The other genera of the subfamily Boleodorinae *sensu* Geraert (2008) apparently have close phylogenetic affinities based on these two ribosomal markers. The GenBank database currently has very few ribosomal sequences of *Boleodorus* (10 SSU sequences, nine of which have been assigned to *B. thylactus* and one to *B. volutus*; 21 LSU sequences, eight of which have been assigned to *B. thylactus*, four to *B. volutus,* and nine unidentified). However, the currently available DNA sequences for the genus formed a maximally supported clade in both phylogenetic trees. Most DNA sequences assigned to *Boleodorus* have not been identified to the species level and their morphological data are not available (see Munawar *et al*., 2021). The new species has close phylogenetic affinity to several sequences of *B. thylactus* in the SSU tree, but its phylogenetic relationships with other available sequences were not resolved in the LSU tree. The identities of several DNA sequences lacking morphological data are under question, and they might represent cryptic species or represent misidentification. Similar to some species under other genera (Aliverdi *et al*., 2022; Sheikhzadeh *et al*., 2022), misassignment of several SSU and LSU sequences assigned to *B. thylactus* was observed in the two presently resolved phylogenies. Before any discussion on the tentative nonmonophyletic status of the species, DNA sequences from the topotype of *B. thylactus* need to be included in future taxonomic studies of Boleodorinae and the morphological data of the sequenced populations must become available.
